# Condições de nascimento e transtorno do déficit de atenção com hiperatividade (TDAH) em adultos nas coortes de nascimento de Pelotas, Rio Grande do Sul, Brasil, de 1982 e 1993 

**DOI:** 10.1590/0102-311XPT138122

**Published:** 2023-10-09

**Authors:** Cid Pinheiro Farias, Pedro San Martin Soares, Fernando C. Barros, Ana Maria Baptista Menezes, Helen Gonçalves, Fernando César Wehrmeister, Ricardo Tavares Pinheiro, Luciana de Avila Quevedo, Bernardo L. Horta

**Affiliations:** 1 Universidade Federal de Pelotas, Pelotas, Brasil.; 2 Universidade Católica de Pelotas, Pelotas, Brasil.

**Keywords:** Transtorno do Déficit de Atenção com Hiperatividade, Parto, Idade Gestacional, Peso ao Nascer, Attention Deficit Disorder with Hyperactivity, Parturition, Gestational Age, Birth Weight, Trastorno por Déficit de Atención con Hiperactividad, Parto, Edad Gestacional, Peso al Nacer

## Abstract

Este artigo avaliou a associação das condições de nascimento com o transtorno do déficit de atenção com hiperatividade (TDAH) em adultos utilizando dados de duas coorte de nascimento da cidade de Pelotas, Rio Grande do Sul, Brasil. Em 1982 e 1993, todos os nascimentos ocorridos na cidade foram identificados e prospectivamente acompanhados. Nos acompanhamentos aos 30 e 22 anos das coortes 1982 (n = 3.574) e 1993 (n = 3.780), respectivamente, os participantes foram examinados e psicólogos treinados aplicaram a *Mini-International Neuropsychiatric Interview* (M.I.N.I.). Aqueles indivíduos que preencheram os critérios diagnósticos do *Manual Diagnóstico e Estatístico de Transtornos Mentais* (DSM-5) foram definidos como positivos para TDAH. A regressão de Poisson com ajuste robusto da variância foi usada para estimar a razão de prevalência (RP) ajustadas para sexo, cor da pele materna, renda familiar, idade materna, escolaridade materna durante a gestação, estado civil materno, paridade e tabagismo materno durante a gestação. A prevalência do TDAH adulto foi de 4,4% e 4,5% nas coortes de 1982 e 1993, respectivamente. A prevalência de TDAH foi maior naqueles que nasceram com menor peso, mas não foi observada tendencia linear. Além disso, aqueles que nasceram com peso entre 3.000 e 3.499 gramas (g) (RP = 1,40, IC95%: 1,05-1,86) apresentaram maior risco para o transtorno. Para a idade gestacional, observamos uma relação inversamente proporcional acerca da presença de TDAH, os pré-termos apresentaram risco 33% maior (IC95%: 0,90-1,96) de ser considerado com TDAH do que os nascidos com 39 ou mais semanas, mas como o intervalo de confiança incluiu a nulidade, essa associação pode ter ocorrido ao acaso. Tais resultados indicam que o peso ao nascer e a idade gestacional podem estar associados ao TDAH adulto.

## Abstract (en)

This study evaluates the association of birth conditions with attention deficit/hyperactivity disorders (ADHD) in adults using data from two birth cohorts in the city of Pelotas Rio Grande do Sul State, Brazil. In 1982 and 1993 all births in the city were identified and have been prospectively monitored. In the follow-ups at 30 and 22 years of the 1982 (n = 3,574) and 1993 (n = 3,780) cohorts, respectively, participants were examined, and trained psychologists applied the *Mini-International Neuropsychiatric Interview* (M.I.N.I.). Those individuals who met the *Diagnostic and Statistical Manual of Mental Disorders* (DSM-5) diagnostic criteria were defined as positive for ADHD. Poisson regression with robust variance adjustment was used to estimate the prevalence ratio (PR) adjusted for sex, maternal skin color, family income, maternal age, maternal schooling during pregnancy, maternal marital status, parity, and maternal smoking during pregnancy. The prevalence of adult ADHD was 4.4% and 4.5% in the 1982 and 1993 cohorts, respectively. The prevalence of ADHD was higher in those born with lower weight, but no linear trend was observed, and those born with weight between 3,000 and 3,499 grams (PR = 1.40; 95%CI: 1.05-1.86) had the highest risk. For gestational age, we observed an inversely proportional relationship for the presence of ADHD: preterm infants had a 33% higher risk (95%CI: 0.90-1.96) of being considered as having ADHD than those born at 39 or more weeks, but as the confidence interval included nullity, this association may have occurred at random. These results indicate that birth weight and gestational age may be associated with adult ADHD.

## Abstract (es)

El presente estudio evaluó la asociación de las condiciones de nacimiento con el trastorno por déficit de atención con hiperactividad (TDAH) en adultos utilizando datos de dos cohortes de nacimiento de la ciudad de Pelotas. En 1982 y 1993 se identificaron todos los nacimientos de la ciudad y se les ha hecho un seguimiento prospectivo. En los seguimientos a los 30 y 22 años de las cohortes de 1982 (n = 3.574) y 1993 (n = 3.780), respectivamente, los participantes fueron examinados y psicólogos capacitados aplicaron la *Mini-International Neuropsychiatric Interview* (M.I.N.I.). Aquellas personas que cumplieron con los criterios de diagnóstico del *Manual Diagnóstico y Estadístico de los Trastornos Mentales* (DSM-5) se definieron como positivos para TDAH. Se utilizó la regresión de Poisson con ajuste robusto de la varianza para estimar la razón de prevalencia (RP) ajustada por sexo, color de piel materna, ingreso familiar, edad materna, educación materna en la gestación, estado civil materno, paridad y tabaquismo materno en la gestación. La prevalencia del TDAH en adultos fue de 4,4% y 4,5 %, en las cohortes de 1982 y 1993, respectivamente. La prevalencia de TDAH fue mayor en aquellos que nacieron con menor peso, pero no se observó una tendencia lineal, y aquellos que nacieron con peso entre 3.000 y 3.499 gramos (RP = 1,40; IC95%: 1,05-1,86) presentaron el mayor riesgo. Para la edad gestacional, se observó una relación inversamente proporcional para la presencia de TDAH, los niños prematuros presentaron un 33 % más de riesgo (IC95 %: 0,90-1,96), de ser considerado como teniendo TDAH que los nacidos con 39 o más semanas, pero como el intervalo de confianza incluyó la nulidad, esa asociación puede haber ocurrido al azar. Tales resultados indican que el peso al nacer y la edad gestacional pueden estar asociados con el TDAH en adultos.

## Introdução

O transtorno do déficit de atenção com hiperatividade (TDAH) é um transtorno do neurodesenvolvimento, de natureza multifatorial ^1^ e sua prevalência em crianças varia entre 4% e 7% ^2^^,^^3^. Em adultos, a proporção de indivíduos com TDAH persistente, isto é, que apresentam os sintomas desde a infância, é de cerca de 2,6%. Enquanto a prevalência de TDAH na idade adulta, independentemente da idade de início dos sintomas, é de aproximadamente 6,8% ^4^.

No que diz respeito aos fatores associados ao desenvolvimento do TDAH, tem sido relatada que a chance de apresentar o transtorno é maior em indivíduos nascidos muito prematuros e com muito baixo peso (*odds ratio* - OR = 2,3; intervalo de 95% de confiança - IC95%: 1,6-3,3) ou extremamente prematuros e com muito baixo peso ao nascer (OR = 4,1; IC95%: 2,4-6,9) ^5^. Além disso, os nascidos considerados pequenos para idade gestacional também estariam sujeitos a um maior risco de apresentar TDAH ^6^^,^^7^. A maioria desses estudos que avaliaram a associação com exposições pré-natais, perinatais e condições de nascimento avaliou o TDAH infantil ^8^. Em adultos, conforme revisão sistemática conduzida por Anderson et al. ^9^, foram identificados apenas dois estudos que analisaram a associação das condições de nascimento com o TDAH. Indivíduos nascidos pré-termo, isto é, antes das 37 semanas de gestação e com muito baixo peso ao nascer apresentaram 3,9 (IC95%: 1,8-8,5) maior chance de serem considerados com TDAH em relação àqueles nascidos com peso normal e a termo. Em outra metanálise, Robinson et al. ^10^ identificaram quatro estudos que avaliaram a associação com TDAH em adultos (≥ 18 anos), em que foram observados um risco 2,4 (IC95%: 1,8-3,1) vezes maior em indivíduos nascidos entre 28 e 31 semanas de gestação e de 2,8 (IC95%: 1,9-4,3) vezes maior naqueles nascidos pré-termo e pequenos para a idade gestacional.

De acordo com o *Manual Diagnóstico e Estatístico de Transtornos Mentais* (DSM-5), o TDAH é considerado um transtorno do neurodesenvolvimento infantil, sendo que um dos critérios para o diagnóstico é a presença de sintomas antes dos 12 anos de idade. Embora o TDAH tenha sido tradicionalmente associado à infância, pesquisas têm demonstrado que uma proporção significativa de indivíduos pode manter o diagnóstico até a idade adulta. Ainda assim, estima-se que apenas cerca de 15% dos indivíduos diagnosticados com TDAH infantil mantenham o diagnóstico aos 25 anos de idade, sugerindo uma expressiva taxa de remissão ^11^. Estudos de revisão e metanálises recentes mostraram a associação entre as condições de nascimento e o TDAH em amostras infantis, porém, evidenciaram uma lacuna na literatura com relação à investigação dessa associação em populações adultas ^9^^,^^10^. Portanto, é possível que a população com TDAH adulto persistente apresente diferenças nos fatores de risco em relação à população com TDAH infantil.

O TDAH em adultos é frequentemente associado a comportamentos de risco e comorbidades psiquiátricas ^12^^,^^13^^,^^14^. Identificar fatores de risco associados ao TDAH nessa população pode contribuir com a identificação de indivíduos que se beneficiarão de intervenções e tratamentos precoces. Assim, este artigo pretende avaliar a associação entre condições de nascimento (peso ao nascer, idade gestacional e crescimento intrauterino) e o TDAH em adultos participantes de duas coortes de nascimento realizadas na cidade de Pelotas, Rio Grande do Sul, Brasil. Nossa hipótese é que o baixo peso ao nascer, a prematuridade e a restrição de crescimento fetal estão associadas a um maior risco de TDAH em indivíduos de 22 e 30 anos de idade.

## Métodos

Este artigo utilizou os dados de duas coortes de nascimento de base populacional e com estratégias de recrutamento semelhantes conduzidas em Pelotas, uma cidade da Região Sul do Brasil. Durante os períodos de 1º de janeiro a 31 de dezembro de 1982 e 1993, equipes treinadas foram designadas para visitar todos os hospitais da cidade diariamente, com o propósito de recrutar mães elegíveis - aquelas residentes na zona urbana do Município de Pelotas - para participar do estudo ^15^^,^^16^. As mães foram entrevistadas, logo após o parto, por meio de um questionário padronizado e pré-codificado. Os partos que ocorreram fora do hospital também foram incluídos nas coortes, uma vez que as mães normalmente procuravam atendimento médico após a gestação e eram recrutadas para o estudo nessa fase.

A coorte de 1982 incluiu 5.914 recém-nascidos, enquanto a de 1993 incluiu 5.249. Esses indivíduos foram prospectivamente acompanhados em diferentes idades. O último acompanhamento da coorte de 1982 foi realizado em 2012, quando os participantes completaram 30 anos de idade. Foram entrevistados 3.701 indivíduos e foram identificados 325 óbitos entre os participantes da coorte, o que representou uma taxa de seguimento de 68,1% ^15^. Já na coorte de 1993, 3.810 participantes foram entrevistados, correspondendo a uma taxa de acompanhamento de 76,3%, após levar em consideração os óbitos identificados entre os participantes ^16^.

### TDAH aos 22 e 30 anos

O TDAH aos 22 e 30 anos nas coortes de 1993 e 1982 foi avaliado por meio do *Mini-International Neuropsychiatric Interview* (M.I.N.I.), versão 5.017, e aplicado por psicólogos treinados. O M.I.N.I. é uma entrevista diagnóstica curta e estruturada, a qual é validada no Brasil ^17^. Os critérios do DSM-5 foram utilizados para definir a presença de TDAH, que incluem ter pelo menos cinco sintomas de desatenção e/ou hiperatividade/impulsividade, com início dos sintomas antes dos 12 anos de idade e sintomas presentes em pelo menos dois ambientes diferentes, como escola e casa, causando prejuízo clinicamente significativo do funcionamento social, acadêmico ou ocupacional por pelo menos seis meses ^18^.

Os critérios do DSM-5 foram utilizados para definir a presença de TDAH, uma vez que o M.I.N.I. abrange os principais sintomas atuais do transtorno ^19^. A adaptação do M.I.N.I. para os critérios do DSM-5 foi realizada ajustando o ponto de corte para a última edição do manual. Dessa forma, foram positivos para TDAH aqueles indivíduos que pontuaram para pelo menos cinco sintomas de desatenção e/ou hiperatividade/impulsividade, com início dos sintomas antes dos 12 anos de idade e, por fim, manifestação de sintomas em pelo menos dois ambientes diferentes, por exemplo, escola, casa, entre outros, causando prejuízo clinicamente significativo do funcionamento social, acadêmico ou ocupacional por pelo menos seis meses ^18^. Essa estratégia tem sido utilizada em estudos prévios ^20^^,^^21^.

### Condições de nascimento

No que diz respeito às condições de nascimento; logo após o parto, os recém-nascidos foram pesados pela equipe do hospital, usando balanças pediátricas, calibradas semanalmente pela equipe da pesquisa, com precisão de 10g. Essa variável, originalmente numérica, foi estratificada em: baixo peso ao nascer (< 2.500g), entre 2.500g-2.999g, entre 3.000g-3.499g e ≥ 3.500g. A idade gestacional foi medida por meio do relato sobre a data da última menstruação (DUM) e classificada como prematuro (< 37 semanas), entre 37-38 e ≥ 39 semanas. O crescimento intrauterino foi medido com ajuda do peso ao nascer, idade gestacional e sexo. Foram definidos como “pequeno para idade gestacional” os indivíduos cujo peso ao nascer, conforme a idade gestacional e sexo, estava abaixo do percentil 10 da população de referência de Williams et al. ^22^.

### Variáveis de confusão

Os seguintes fatores de confusão foram considerados: sexo; cor da pele materna; renda familiar em salários mínimos; idade materna em anos completos; escolaridade materna na gestação em anos completos; estado civil materno; paridade; e tabagismo materno na gestação. Esses dados foram coletados nos inquéritos perinatais das coorte de 1982 e 1993 durante as entrevistas realizadas com as mães, logo após o parto.

Para avaliar o tabagismo materno durante a gestação, na coorte de 1982, as mães foram questionadas sobre a categoria de consumo de cigarros durante a gravidez que se enquadrava em: não fumou 1-14 cigarros por dia durante parte da gravidez; fumou 1-14 cigarros por dia durante toda a gravidez; fumou ≥ 15 cigarros por dia durante parte da gravidez; e fumou ≥ 15 cigarros por dia durante toda a gravidez). Já na coorte de 1993, o tabagismo materno durante a gestação foi avaliado de forma mais detalhada, questionando as mães sobre a quantidade diária de cigarros fumados e o número de dias em que isso ocorreu em cada trimestre da gestação. Nesta pesquisa, as mães que relataram o consumo de cigarros em qualquer momento da gestação foram categorizadas como positiva para fumo na gestação.

### Análise estatística

Foi apresentada a prevalência do TDAH, bem como das demais variáveis de interesse, juntamente com seus valores absolutos. Foram realizados testes de qui-quadrado para comparar a distribuição das variáveis peso ao nascer, a idade gestacional e o crescimento intrauterino no período perinatal com as dos acompanhamentos aos 22 e 30 anos, a fim de determinar se as perdas de seguimento poderiam afetar as amostras.

Foi utilizada a regressão de Poisson com ajuste robusto da variância para estimar a razão de prevalência (RP) bruta e ajustada de TDAH adulto, agregando os dados das duas coortes, de acordo com o peso ao nascer, a idade gestacional e o crescimento intrauterino. Os modelos de regressão foram aplicados separadamente para cada uma das variáveis de exposição de interesse. A análise ajustada incluiu os fatores de confusão descritos acima e uma variável que indicava a coorte do participante. Para as variáveis ordinais foi calculado o valor de p de heterogeneidade e de tendência linear e aquele com menor valor foi apresentado. Ainda na análise multivariada, foi aplicado o teste de razão de verossimilhança para avaliação da possível interação da coorte com as associações de interesse. Foi estabelecido um valor de p < 0,05 para a obtenção da significância estatística e todas as análises foram realizadas usando Stata, versão 15.0 (https://www.stata.com).

### Considerações éticas

Os acompanhamentos das coortes de 1982 e 1993, aos 30 e 22 anos, foram aprovados pelo Comitê de Ética em Pesquisa da Faculdade de Medicina da Universidade Federal de Pelotas (sob os registros 4.06.01.087 e 1.250.366, respectivamente). Em ambas coortes, as avaliações foram somente realizadas após a assinatura do Termo de Consentimento Livre Esclarecido (TCLE).

## Resultados

Neste artigo, foram incluídos 7.354 participantes das duas coortes. A amostra analisada da coorte de 1993 apresentou diferenças na distribuição do crescimento intrauterino em relação à coorte original (Material Suplementar: https://cadernos.ensp.fiocruz.br/static//arquivo/suppl-e00138122_1635.pdf). A Tabela 1 mostra que nas duas coortes a proporção de mulheres avaliadas na idade adulta foi discretamente maior e mais da metade dos participantes nasceram em famílias com renda menor ou igual a três salários mínimos. No tocante a escolaridade materna, observamos um aumento nos anos de escolaridade completos entre as duas coortes. Apesar disso, em 1993, 26,4% das mães apresentavam quatro ou menos anos de escolaridade. No que diz respeito às condições de nascimento, as prevalências de baixo peso (< 2.500g) e prematuridade (< 37 semanas) aumentaram de 7,2% para 9% e de 5,6% para 10,5%, respectivamente, enquanto o retardo de crescimento intrauterino diminuiu de 14,2% para 9,3%. A prevalência total de TDAH aos 22 e 30 anos foi de 4,5% e 4,4%.

**Tabela 1:** Distribuição da população estudada segundo critérios socioeconômicos, demográficos, condições de nascimento e prevalência de transtorno do déficit de atenção com hiperatividade (TDAH). Coorte de nascimentos em Pelotas, Rio Grande do Sul, Brasil, 1982 e 1993.

Variáveis	Coorte de 1982 (%)	Coorte de 1993 (%)
Sexo		
Feminino	52,0	53,4
Masculino	48,0	46,6
Cor da pele materna *		
Branca	76,1	76,5
Outras	24,1	23,5
Idade da mãe no nascimento (anos) *		
< 20	15,0	17,4
20-29	57,4	53,4
≥ 30	27,7	29,3
Escolaridade materna (anos) *		
0-4	32,1	26,4
5-8	43,1	46,9
9-11	11,0	18,5
≥ 12	13,8	8,1
Estado civil materno *		
Com companheiro(a)	92,3	88,0
Sem companheiro(a)	7,7	12,0
Renda familiar (salários mínimos) *		
≤ 1	19,5	17,7
1,1-3	49,5	41,5
3,1-6	19,5	24,9
> 6	11,5	16,0
Paridade *		
Nulípara	40,2	35,6
1-2	44,6	45,6
≥ 3	15,3	18,8
Fumo materno durante a gravidez (sim)	35,3	32,6
Baixo peso ao nascer *	7,2	9,0
Idade gestacional (DUM) *		
< 37	5,6	10,5
37-38	22,2	20,0
≥ 39	72,2	69,4
Pequeno para idade gestacional *	14,2	9,3
Prevalência de TDAH	4,4	4,5
N *	3.574	3.780

DUM: data da última menstruação.

* Cor de pele materna na coorte de 1982 (n = 3.573) e na coorte de 1993 (n = 3.778); baixo peso ao nascer na coorte de 1982 (n = 3.573) e em 1993 (n = 3.775); idade gestacional na coorte de 1982 (n = 2.882) e em 1993 (n = 3.390); pequeno para idade gestacional na coorte de 1982 (n = 2.880) e em 1993 (n = 3.725); renda familiar em salários mínimos na coorte de 1982 (n = 3.557) e em 1993 (n = 3.711); idade materna no nascimento na coorte de 1982 (n = 3.573) e em 1993 (n = 3.780); estado civil materno na coorte de 1982 (n = 3.570); paridade na coorte de 1982 (n = 3.573); escolaridade materna na coorte de 1982 (n = 3.569) e em 1993 (n = 3.775). Foi omitido o número amostral das variáveis que contaram com o número total da amostra.

A Tabela 2 apresenta os resultados das análises agregando os dados das duas coortes, uma vez que as associações entre condições de nascimento e TDAH foram similares entre as coortes (p de interação > 0,51). A associação entre peso ao nascer e TDAH adulto não apresentou uma magnitude de associação linear, sendo que o maior risco foi observado naqueles que nasceram com peso entre 3.000g e 3.499g (RP = 1,40; IC95%: 1,05-1,86), seguidos por aqueles indivíduos que nasceram com peso entre 2.500-2.999g, que apresentaram um risco 37% maior em relação ao grupo referência (RP = 1,37; IC95%: 1,00-1,87). Por outro lado, para a idade gestacional, a associação foi inversamente proporcional, mas não podemos descartar que esta associação tenha sido devido ao acaso (p = 0,10). No tocante ao crescimento intrauterino, os indivíduos que nasceram pequenos para a idade gestacional apresentavam menor risco de TDAH na idade adulta, mas, como o intervalo de confiança incluiu a nulidade, também não é possível descartar que esse resultado seja decorrente do acaso. Em todas as análises, a magnitude das associações apresentou pequena diminuição após ajuste para os fatores de confusão.

**Tabela 2:** Análise de prevalência, razão de prevalência (RP) da associação bruta e ajustada de condições de nascimento de transtorno do déficit de atenção com hiperatividade (TDAH) em adultos aos 22 e 30 anos da coorte de nascimentos de 1982 e 1993 de Pelotas, Rio Grande do Sul, Brasil (n = 7.354).

Variáveis	Prevalência de TDAH (%)	RP			
		Bruta (IC95%)	Valor de p	Ajustada * (IC95%)	Valor de p
Peso ao nascer (g)			0,10		0,16
< 2.500	4,4	1,25 (0,81-1,94)		1,19 (0,75-1,89)	
2.500-2.999	4,8	1,37 (1,01-1,86)		1,37 (1,00-1,87)	
3.000-3.499	4,9	1,41 (1,07-1,86)		1,40 (1,05-1,86)	
≥ 3.500	3,5	1,00		1,00	
Idade gestacional (semanas)			0,05		0,10
< 37	5,6	1,34 (0,92-1,96)		1,33 (0,90-1,96)	
37-38	5,2	1,24 (0,94-1,62)		1,16 (0,88-1,53)	
≥ 39	4,2	1,00		1,00	
Pequeno para idade gestacional			0,21		0,13
Sim	3,6	0,78 (0,53-1,15)		0,74 (0,50-1,10)	
Não	4,6	1,00		1,00	

IC95%: intervalo de 95% de confiança.

* Regressão de Poisson foi usada para sexo, cor da pele materna, renda familiar, idade materna, escolaridade materna na gestação, estado civil materno, paridade e tabagismo materno na gestação.

## Discussão

Nosso estudo observou que indivíduos nascidos com peso menor do que 3.500g apresentavam maior risco para o TDAH. Além disso, observamos tendência a maior risco de TDAH naqueles que nasceram com menor idade gestacional, mas não podemos descartar que essas associações tenham ocorrido por acaso. Por outro lado, os indivíduos nascidos pequenos para a idade gestacional apresentavam tendência a menor risco de serem considerados como tendo TDAH. O peso ao nascer está negativamente relacionado com a idade gestacional e o crescimento intrauterino ^23^, os nossos resultados sugerem que a associação observada entre peso ao nascer e o TDAH seja melhor explicada pela menor duração da gestação, uma vez que a menor idade gestacional esteve associada com uma tendência de maior risco para TDAH.

No que diz respeito ao maior risco de TDAH em indivíduos que nasceram com peso menor, apesar da associação não ter sido linear, os nossos resultados são condizentes com o que vem sendo observado na literatura ^5^^,^^24^. Com relação à idade gestacional, outros estudos também observaram maior risco de TDAH na idade adulta em indivíduos que nasceram pré-termo ^7^^,^^10^. Por outro lado, no tocante ao crescimento intrauterino, Halmøy et al. ^25^ observaram um risco 60% (IC95%: 1,0-2,5) maior para a persistência TDAH em adultos nascidos pequenos para idade gestacional. Enquanto a metanálise realizada por Robinson et al. ^10^, utilizando dados de oito coortes, encontrou um risco 2,8 (IC95%: 1,85-4,25) vezes maior para o TDAH em adultos nascidos pequenos para idade gestacional, independentemente da idade. Outro estudo observou uma pontuação média mais alta para sintomas de desatenção ou hiperatividade quando comparados aos nascidos com tamanho normal ^26^. Neste estudo, não observamos a mesma associação, inclusive quando consideramos o número de sintomas de desatenção e ou hiperatividade.

As condições de nascimento têm sido usadas como um indicador da qualidade das condições do ambiente intrauterino e apresentam consequências em longo prazo sobre a saúde do indivíduo, tais quais maiores riscos de doenças crônicas respiratórias, cardíacas, renais, sistema endócrino e psiquiátricas ^27^^,^^28^^,^^29^.

Os mecanismos pelos quais as condições de nascimentos contribuem para o desenvolvimento do TDAH ainda são pouco compreendidos. A menor duração da gestação levaria a interrupção no neurodesenvolvimento fetal, sobretudo por conta do último trimestre da gravidez ser um período bastante crítico para os processos de organização neuronal e neurogênese. El Marroun et al. ^30^ observaram que a duração da gestação está positivamente associada com o volume cerebral aos 10 anos e menores volumes das estruturas frontais, parietais e cerebelares foram relatados em estudos clínicos em crianças com TDAH ^31^^,^^32^, estando essas alterações morfométricas correlacionadas aos défices funcionais, como de atenção, controle inibitório e de memória de trabalho ^33^^,^^34^. No que diz respeito ao peso ao nascer, Pascoe et al. ^35^ observaram em uma coorte de nascimentos que o peso foi positivamente correlacionado com o volume de substância cinzenta e perfusão em regiões corticais e subcorticais, em especial nos córtices frontal, temporal, parietal, occipital, giro caudado esquerdo, tálamo e hipotálamo em adultos com idade entre 27 e 29 anos. Alterações que correspondem tanto ao que vem sendo observado em estudos de neuroimagem em indivíduos com TDAH, quanto com o que tem sido observado do ponto de vista funcional ^36^^,^^37^. Tais alterações foram associadas à qualidade da memória de trabalho, à atenção e ao controle inibitório, além de vias colinérgicas e aminérgicas, no caso, associadas aos sintomas e tratamento farmacológico do TDAH em adultos ^38^.

Com relação aos pontos positivos deste artigo, a coleta das informações sobre as condições de nascimento foi feita imediatamente após o nascimento, garantindo a qualidade das informações obtidas e reduzindo a possibilidade de ocorrência de erro de mensuração. As variáveis de confusão foram medidas logo após o nascimento, próximo a sua ocorrência, minimizando a possibilidade de confusão residual. A avaliação do TDAH foi realizada por psicólogos treinados, o que também reduziu a possibilidade de erro de classificação. Além disso, esta pesquisa apresentou um poder estatístico superior a 80%. Entre os pontos fracos, pode-se apontar que o diagnóstico de TDAH foi coletado aos 30 e 22 anos, utilizando um teste de rastreio contendo componentes recordatórios, sendo suscetível a vieses de informação, aumentando o risco de a prevalência ser subestimada.

Esta pesquisa observou um maior risco para o TDAH adulto em nascidos com menor tempo de gestação ou com baixo peso utilizando dados agregados de duas coortes de nascimentos em um acompanhamento de 22 e 30 anos. Embora os resultados tenham alcançado a significância estatística somente para o peso ao nascer, também observamos um maior risco para o TDAH adulto em adultos nascidos pré-termo, sendo maior que 30% naqueles nascidos com menos de 37 semanas de gestação. Deve-se levar em conta que a baixa prevalência do nosso desfecho implica na redução do poder estatístico. Nossos resultados reforçam a importância do neurodesenvolvimento fetal sobre a saúde mental em longo termo, conforme vem sendo observado em estudos similares, destacando a importância sobre o cuidado e acompanhamento pré-natal e seu impacto sobre a saúde mental. Além disso, este artigo utilizou dados que representam uma população adulta de um país em desenvolvimento, ainda escassos na literatura.
